# Supplementary material for: Development of 3D Cell-Based Fluorescent Reporter Assay for Screening of Drugs Downregulating Telomerase Reverse Transcriptase
Source: Bioengineering (Basel). 2025 Mar 23;12(4):335. doi: 10.3390/bioengineering12040335 (PMC12024458; doi:10.3390/bioengineering12040335)
Supplement: Supplementary file 1 [file bioengineering-12-00335-s001.zip › bioengineering-3509815-supplementary.pdf]

Supplementary Materials

Development of 3D Cell-Based Fluorescent Reporter Assay for Screening of Drugs Downregulating Telomerase Reverse Transcriptase

You Li, Fengli Zhang, Zhen Qin and Shang-Tian Yang *

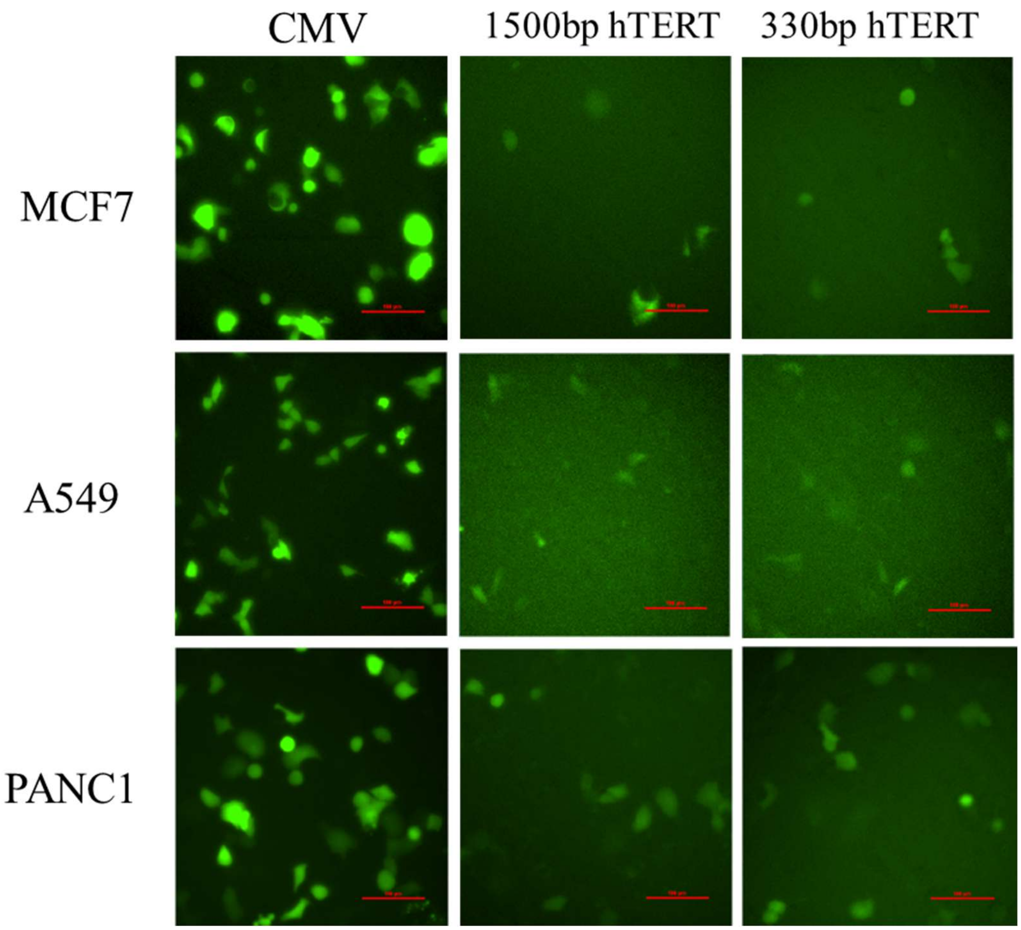

Figure S1. Fluorescent microscopic images of MCF7, A549 and PANC1 cells 24 h after transfection with plasmids for transient expression of EGFP under the control of CMV, 1500 bp hTERT, and 330 bp hTERT promoters, respectively. Images were taken at 480 ± 30 nm excitation and 535 ± 40 nm emission. (Scale bar: 100 µm)

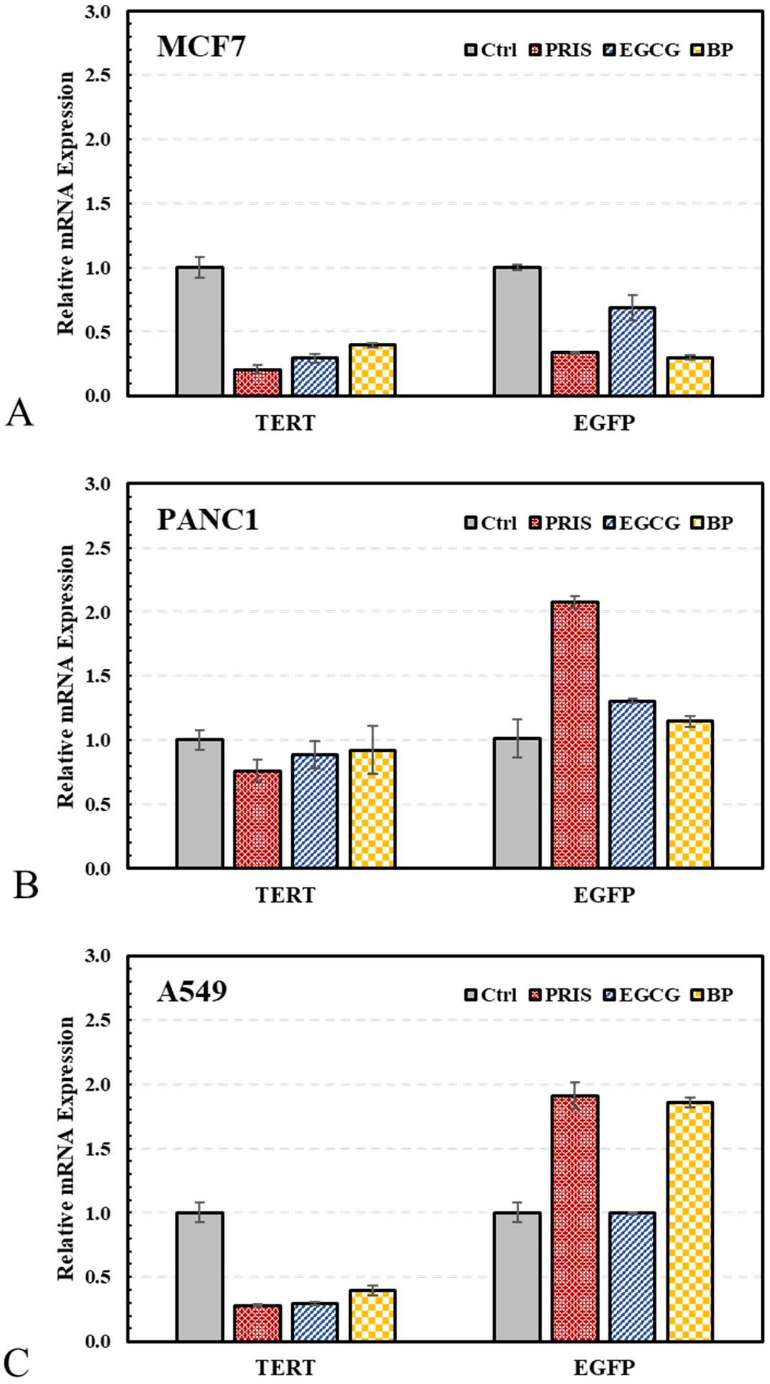

Figure S2. Comparison of EGFP and hTERT mRNA levels in engineered cells 48 h after treatment with 1.5 μM pristimerin (PRIS), 200 μM EGCG, and 80 μg/mL BP, respectively. The relative expression levels are reported with the control (no drug treatment) as 1. (A) MCF7-hTERT-EGFP, (B) PANC1- hTERT -EGFP, (C) A549-hTERT -EGFP.

Table S1. Drug regulation effect on hTERT expression observed in 3D assay, mRNA test, and prior studies

Drug	Category	Mechanism	3D assay	mRNA test	Prior studies
Pristimerin	Natural triterpenoid	Regulate signaling pathways (MAPK, PI3K/AKT/mTOR, etc.) (Li et al., 2019))	↓	↓	↓ (Liu et al., 2015; Deeb et al., 2017)
Doxorubicin	Antibiotic	Intercalate into DNA-DNA pairs, inhibit topoisomerase II (Tacar et al., 2012)	↓	↓	↓ (Eskiocak et al, 2008; Lanvers-Kaminsky et al. 2005)
Cisplatin	Platinum-based molecule	Crosslink with the urine bases, prevent DNA repair (Dasari & Tchounwou, 2014)	↓	↓	↓(Lee et al., 2007)<br>↑ (Guo et al., 2009)<br>– (Zhang et al., 2002)
Blasticidin	Antibiotic	Block protein synthesis by binding to the ribosomal P-site (IzuMi et al., 1991, Svidritskiy et al., 2013)	↓	↓	NA
Tamoxifen	Targeted drug	Target estrogen receptor (Mandlekar, 2001)	↑	↓	↓(Wang et al., 2002; Zhou et al., 2013)
Paclitaxel	Natural diterpene alkaloid	Target tubulin, disrupt mitotic spindle assembly (Barbuti & Chen, 2015)	–	–	– (Hanna et al., 2012; Shafer et al., 2009)

↑: upregulation; ↓: downregulation; –: no effect
